# Supplementary material for: Equine Genital Squamous Cell Carcinoma Associated with EcPV2 Infection: RANKL Pathway Correlated to Inflammation and Wnt Signaling Activation
Source: Biology (Basel). 2021 Mar 21;10(3):244. doi: 10.3390/biology10030244 (PMC8003831; doi:10.3390/biology10030244)
Supplement: Supplementary file 1 [file biology-10-00244-s001.zip › Supplementary_files/Table S1.docx]

**Table S1.** Viral DNA assessment on control samples.

| Control ID | DNA | |
| --- | --- | --- |
|  | *B2M* | *E6* |
| P1 | 30.63±0.13 | 0.00 |
| P2 | 29.65±0.16 | 0.00 |
| P3 | 28.16±0.11 | 0.00 |
| P4 | 28.96±0.10 | 0.00 |
| P5 | 28.18±0.06 | 0.00 |
| V1 | 27.78±0.06 | 0.00 |
| V2 | 31.43±0.21 | 0.00 |
| V3 | 29.17±0.03 | 0.00 |
| V4 | 29.92±0.14 | 0.00 |
| V5 | 29.46±0.06 | 0.00 |
