# Supplementary material for: Equine Genital Squamous Cell Carcinoma Associated with EcPV2 Infection: RANKL Pathway Correlated to Inflammation and Wnt Signaling Activation
Source: Biology (Basel). 2021 Mar 21;10(3):244. doi: 10.3390/biology10030244 (PMC8003831; doi:10.3390/biology10030244)
Supplement: Supplementary file 1 [file biology-10-00244-s001.zip › Supplementary_files/Figure S1.pdf]

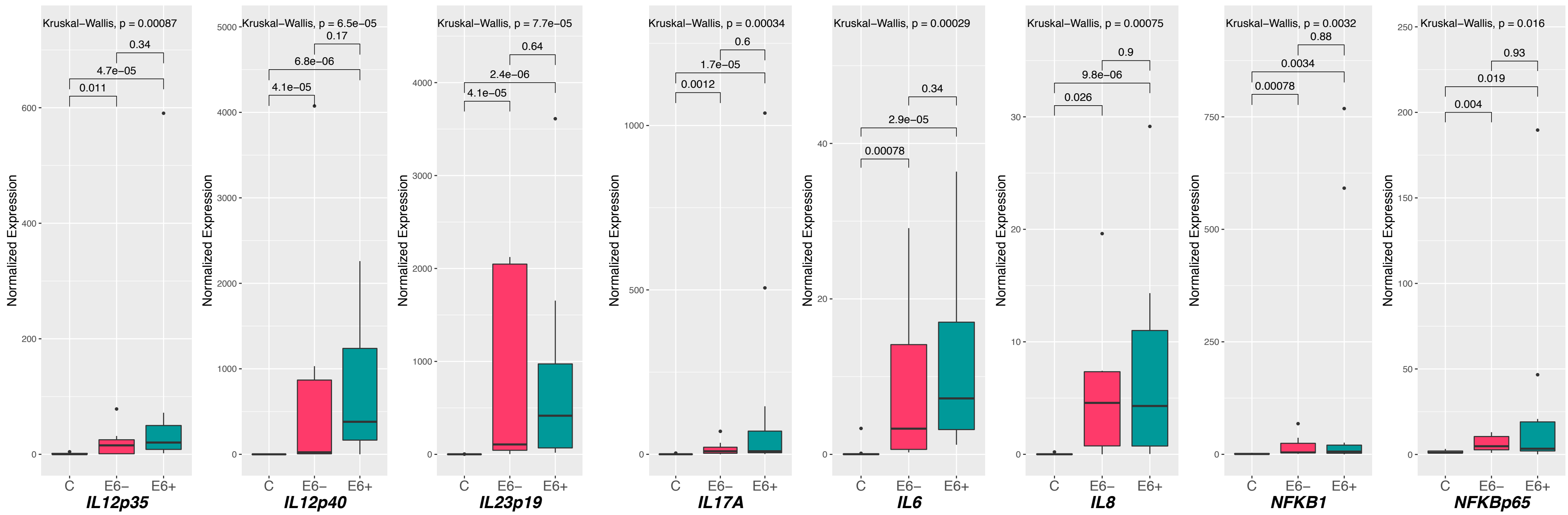

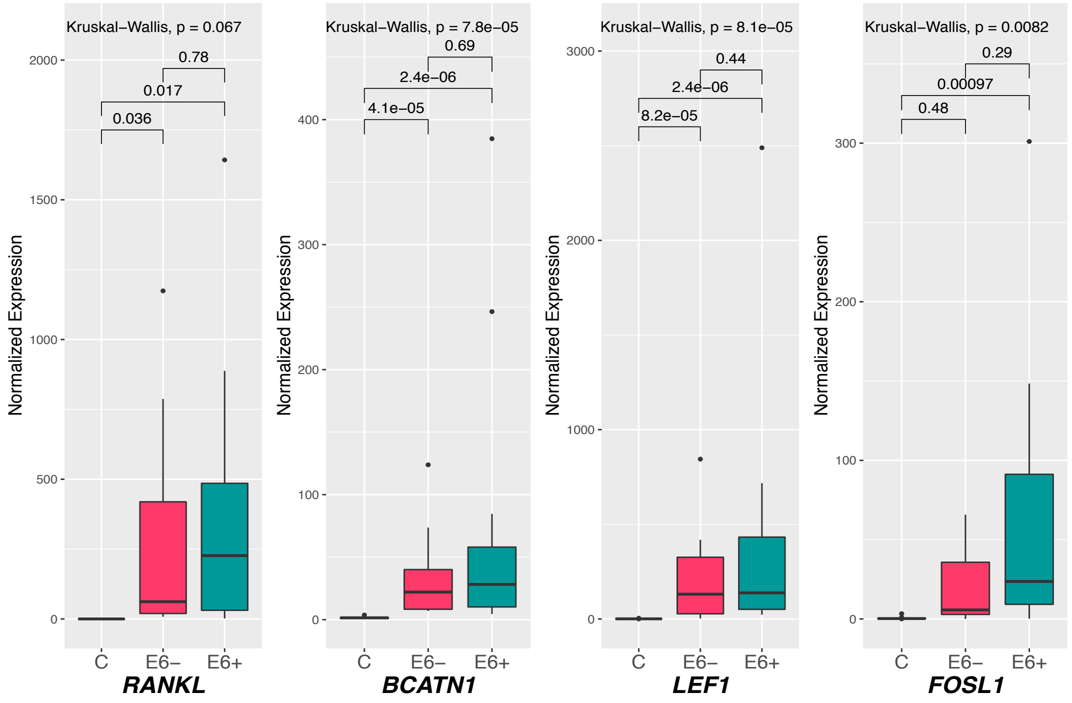

Figure S1. Box-plot of IL12p35, IL12p40, IL23p19, *IL6*, *IL8*, *NFKB1*, *IL17A*, *NFKBp65*, *RANKL*, *LEF1*, *BCATN1* and *FOSL1* gene expression comparing control E6- and E6+ samples. The statistical significance (P<0.05) of median differences was assessed through Kruskal-Wallis test after normalizing expression levels whit those of the reference *B2M*. Kruskal-Wallis significance is reported in the upper part of the graph and the statistically significant values among each comparison are shown on the horizontal bars. C: Control group; *E6-*: samples not expressing *E6*; *E6+*: samples expressing *E6* gene.
